# The Role of Perceived Unjust Treatment in Unmet Needs for Primary Care Among Finnish Roma Adults

**DOI:** 10.3390/ijerph17165825

**Published:** 2020-08-12

**Authors:** Riikka Lämsä, Anu E. Castaneda, Anneli Weiste, Marianne Laalo, Päivikki Koponen, Hannamaria Kuusio

**Affiliations:** 1 Faculty of Medicine, University of Helsinki, 00014 Helsinki, Finland; 2 Finnish Institute for Health and Welfare, 00271 Helsinki, Finland; anu.castaneda@thl.fi (A.E.C.); Anneli.weistepaakkanen@gmail.com (A.W.); Marianne.laalo@thl.fi (M.L.); Paivikki.koponen@thl.fi (P.K.); hannamaria.kuusio@thl.fi (H.K.)

**Keywords:** unmet need, unjust treatment, healthcare services, minorities, Roma

## Abstract

The main goal in developing services is to guarantee equal access to healthcare services that are suited to the patients’ needs. Previous studies have shown that the Roma are more likely to experience unjust treatment in health services than the general population. This study examines the association between perceived unjust treatment in healthcare and self-assessed unmet need for primary care provided by general practitioners (GPs) and nurses among the Finnish Roma. The data from the Finnish Roma Wellbeing Study (Roosa), conducted in 2017–2018, were used. Snowball sampling was used in recruiting study participants (*n* = 365, 61% women). Logistic regression was used to test the association between perceived unjust treatment and unmet need for primary care. Confounders used were gender, age, marital status, education, employment, and self-rated health. Those who had experienced unjust treatment in healthcare were more likely to report unmet need for care provided by GPs (odds ratios (OR) = 6.44; *p* < 0.001) and nurses (OR = 11.18; *p* < 0.001) than those who felt that they had been treated justly. This association remained after adjustments for the confounders. Bidirectional guidance between the Roma and service providers should be improved and the Roma communities involved in service development using participatory methods.

## 1. Introduction

In Finland, as in many Western countries, equity in health and equal access to healthcare are the main goals of social and health policies, as well as service development [1]. However, in many European countries, the Roma suffer from poor access to healthcare, which leads to high levels of unmet needs for healthcare (e.g., [2,3,4]). According to previous studies, the Roma experience a considerable amount of discrimination throughout Europe [5,6]. In Finland, discrimination and perceived unjust treatment in public services have also been shown to be common among the Finnish Roma, and other minorities, such as the foreign-born population [7,8]. In general, perceived discrimination has been linked to increased psychological distress [9], lower life satisfaction [10], and increased experiences of unmet needs for healthcare [11]. 

### 1.1. The Finnish Roma as Ethnic Minority Group in Finland

Finnish Roma—along with the Sámi and Finnish Swedes—form one of the largest linguistic and cultural minorities in Finland. According to official Finnish estimates, there are about 10,000–12,000 Finnish Roma people living in Finland. Although there are Finnish Roma communities throughout the country, the majority of Finnish Roma live in the cities of southern and western Finland [12]. Finnish Roma have lived in Finland since the 1500s. From the end of the nineteenth to the beginning of the twentieth century, the official policy was to assimilate the Finnish Roma into the Finnish population. Finnish Roma children were frequently separated from their parents and their Kale language was banned. Up until the 1960s, the Finnish Roma still had a travelling lifestyle. The Finnish policies concerning minorities started to change in the 1970s. The new goal was to integrate the Finnish Roma into Finnish society whilst still respecting their wish to maintain a distinct identity [13]. In the 1970s, the living conditions of the Finnish Roma were examined in a census study that showed their living conditions and socio-economic background to be much weaker than those of the general population [14]. The study laid the foundation for the state’s contribution in accommodating the Finnish Roma in public sector housing [15]. In the period between the 1970s and 1990s, there was much activity in social, education, and cultural policies concerning the Roma. This activity led to the social awakening of the Finnish Roma and to the development of the Kale language. The Finnish Roma were, for instance, given the right to study Kale as a mother tongue in schools [16].

The culture of the Finnish Roma is patriarchal. It is believed that the man is the head of the family and the woman is the heart. This means that while women have the main responsibility for household management and childcare. The man’s task is to support the family and to be the representative for the family outside the home. Nowadays, the roles are changing, and women more often educate themselves and work outside the home [12]. 

Today, the Finnish Roma enjoy full civil rights. Even though the social situation of the Finnish Roma has improved, their status is not yet equal to that of the general population in Finland. For instance, it is still hard for the Roma to obtain accommodation in the private housing market [17]. On average, the Roma are in a weaker economic and social position than other Finnish people [18]. They suffer from a variety of health problems and have a high prevalence of self-reported chronic diseases (as well as their risk factors). Nearly one in three men and one in four women over the age of 55 reported that they suffered from coronary heart disease. Diabetes rates were also high: one-fourth of men and nearly one-third (29%) of women reported having diabetes. [19]. Moreover, the Finnish Roma seem to have a high prevalence of mental health problems. Depression, diagnosed or treated by a doctor within the past 12 months, was reported by 14% of the men and 16% of the women [7]. Health-endangering lifestyle choices, such as smoking or a lack of physical exercise, are common among both men and women [20,21]. There is no information about the Finnish Roma that could be comparable to the general population since a random sample of an ethnic minority is not possible to obtain due to legal reasons.

### 1.2. Primary Healthcare System in Finland

In Finland, healthcare is mostly funded publicly, and the responsibility for providing healthcare services is devolved to the (around) 300 municipalities. Primary healthcare is provided by health centres, established either by a single municipality or jointly by two or more neighbouring municipalities. The municipalities have also the right to buy services from the private sector. Health centre services include medical consultations, dental care, preventive care, and environmental healthcare. Nurse appointments are common, and in many cases (e.g., on-call visits), nurses act as gatekeepers to the general practitioners (GPs). In some health centres, the patient can make an appointment directly with the general practitioner (GP). In Finland, only doctors can prescribe medication or refer patients to further treatment in specialized care services. In addition to public health services, the private healthcare sector provides healthcare services. In addition, employees are covered by occupational healthcare—the scope and extent of which varies from one employer to another [22].

All residents, including the Finnish Roma, are covered by the National Health Insurance (NHI) system in Finland. Thus, the Finnish Roma have equal rights to care, but not enough reliable research evidence exists on how the Finnish Roma healthcare usage compares to the general population. A study observed that among the Finnish Roma, 73% of men and 85% of women had had at least one general practitioner (GP) visit during the past twelve months [23]. In 2017, nearly 70% of the general population had had GP visits [24].

### 1.3. Unmet Need for Healthcare 

Unmet healthcare needs is a multi-interpretative concept that can be defined, for example, from the individual, the clinician, or the healthcare system’s point of view. An individual-level subjective unmet need is perceived when an individual experiences a need for healthcare services but cannot obtain an effective treatment that could improve his or her health. Unmet needs can also be unperceived in the cases in which an individual does not perceive that he or she needs healthcare services (for example hypertension without symptoms). A person’s subjective experience of a need for healthcare may also be influenced by a healthcare professional’s objective assessment of the need for healthcare. Clinician-validated unmet healthcare needs arise when an individual does not receive the treatment that a clinician judges appropriate. In some cases, the unmet healthcare need can be acceptable from the point of view of the healthcare system or the whole society based on, for instance, a lack of resources. In these cases, there is a risk of increase in inequality if the unmet healthcare needs become systematically related to socioeconomic or other personal characteristics [25,26,27]. 

Several studies have identified specific population groups with increased likelihood of reporting an unmet need for healthcare; these groups include women, people already suffering from poor health, people with lower income, the unemployed, and various ethnic groups [28,29,30,31,32]. In many European countries, the Roma people have a significantly worse health and education profile, and their unemployment rate is higher than non-Roma people [33]. A Spanish study has shown that Roma women are more likely to suffer from obesity, depression, and migraines than non-Roma women [34]. They also use more alcohol and are less likely to get regular smear tests and mammograms.

The Roma are discriminated against across Europe. One way of defining discriminatory experiences is as unequal or unjust treatment of individuals or a defined group [35]. There are many forms of discrimination and unjust treatment: direct discrimination is the naming or intimidation of an individual or group, while indirect discrimination includes seemingly equal rules, practices, and treatment that, in truth, are applied unfairly and discriminatingly based on a personal characteristic. Indirect discrimination may be either conscious or unconscious. Ethnic discrimination or unjust treatment can be related to characteristics such as an atypical appearance or clothing. The discrimination in service settings can mean exclusion from certain services, poorly implemented counselling and guidance, or ignoring the needs of the discriminated [36]. Discrimination and perceived unjust treatment influence health-related behaviour, including the use of health services, by decreasing the trust in healthcare professionals and diminishing the social, emotional, and physical resources [37,38,39].

Several studies have shown that the Roma are discriminated against [5,7,40] and their access to healthcare is somewhat limited [2,3,4]. There are no significant Roma studies to be found on the subject of discrimination and unjust treatment and their association with unmet needs for primary care provided by GPs and nurses. No previous studies have been done in Finland concerning the factors associated with unmet need for healthcare among the Finnish Roma. This study aims to partly fill this gap by examining (1) the prevalence of the self-assessed need for primary care, unmet needs for primary care, perceived unjust treatment in healthcare, and the self-reported health among the Finnish Roma; and (2) the association between perceived unjust treatment in healthcare settings and self-reported unmet needs for primary care provided by GPs and nurses among the Finnish Roma in Finland. The study also takes into account the potential effects of various confounding factors, such as gender, age, marital status, education, employment status, and self-perceived health. 

## 2. Data and Method

This study uses data collected in the Finnish Roma Wellbeing Study (Roosa), conducted in Finland (2017–2018). A total 365 (of which 61% were female) participated in at least one part of the study. The Roosa study utilized a culture-sensitive and participatory study design (CBPR). In the CBPR approach, community members and academic researchers work in partnership in each step of the research process to maximize the input from the community members [41,42]. The Finnish Roma participated in the study, not only as respondents, but also as employees who recruited participants to the study and as experts. Four Finnish Roma persons acted as regional coordinators during the study. They also acted as cultural interpreters between the Roma community and the researchers, informed the local Roma people about the study, and recruited participants through their personal contacts and by organizing survey-related events. In addition, representatives from several Roma non-government organizations (NGOs) were involved in planning the research design, interpreting the results from a cultural viewpoint, and writing and commenting on the study report. The results of this article regarding unjust treatment were discussed with the Finnish Roma representatives. Due to COVID-19 measurements, we were not able to interview, but send an e-mail to them (date 15 May 2020), and their outlook on the results is presented in the results [43]. 

The Roosa study methods and the contents of the survey were based on previous Finnish health examination surveys for both the general population and minority groups (e.g., [44,45]). The feasibility of the data collection methods was tested in a pilot-study [46]. However, the recruitment of participants confronted difficulties and, consequently, the earlier sample size and power calculations with target numbers of men/women, as well as the size of different age-groups and study areas, were not feasible. Snowball sampling [47] was used to recruit as many participants as possible in the determined study period due to fact that a random sample was not possible to obtain. One aim of the recruitment process was to include persons from all socioeconomic backgrounds, regions of Finland, and age groups in the study; however, there is no conclusive way of knowing how the respondents represent the rest of the Finnish Roma population. 

The Roosa study data collection included a health examination made by a research nurse as well as a structured face-to-face interview or a self-filled questionnaire. There were two versions of the questionnaire/interview, a long and a short version. Of the all respondents, 68% filled the long version of the questionnaire while 31% filled the short version. Five persons participated only in the health examination. The short version was answered by those who, due to a lack of time, a lack of functional capacity, or other reasons, could not respond to the longer version. There were participants from all parts of Finland, but the regional distribution cannot be described in more detail due to data protection concerns. 

The current study uses data from the structured interviews and self-administered questionnaires; the health examination data is not included in the current study.

Ethical approval was obtained from Coordinating Ethics Committee of the Helsinki University Hospital (HUS/1445/2016). All participants signed an informed consent for a health examination. 

### Measures

In this study, the two outcome measures for unmet need for primary care provided by a GP and unmet need for primary care provided by a nurse was based on the respective questions in the survey: ‘Do you feel that you have adequately received primary care services provided by a GP during the past 12 months?’ and ‘Do you feel that you have adequately received primary care services provided by a nurse during the past 12 months?’. Both questions were answered by choosing one of four options: (1) ‘I have not needed it’; (2) ‘I would have needed it, but did not receive the service’; (3) ‘I have used the service but it was not adequate’; and (4) ‘I have used the service and it was adequate’. Those who did not need the services were excluded from the analyses. The variable ‘need for services’ included the response options 2, 3, and 4, while response options 2 and 3 were used to measure the unmet needs for healthcare services.

Perceived unjust treatment in healthcare settings was assessed using the question: ‘Have you visited a hospital, a health centre or other health-related services and been in contact with a doctor, a nurse or other health professional?’ Here, the response format included options (1) ‘No’; (2) ‘Yes, and I was treated justly’; and (3) ‘Yes, and I was treated unjustly’.

The variable self-rated health was formed using the respondent answers to the question: ‘How would you describe your current health status?’ The respondents rated their health on a five-item Likert-scale ranging from 1 (good) to 5 (poor). A binary transformation was used in the analysis, combining the options from 3 to 5 as ‘moderate or lower’ and options 1 and 2 as ‘good or rather good’. 

Potential confounders included respondents, age, gender, marital status, highest obtained education and, in the case of 18–64-year-old respondents, self-reported employment status for (1 = ‘employed’; 2 = ‘unemployed’; and 3 = ‘others’). 

The prevalence of need for primary care provided by GPs and nurses, of unmet need for primary care for GPs and nurses, of unjust treatment in healthcare, and of self-rated health was calculated separately for men and women.

Logistic regression analyses were used to test the association of perceived unjust treatment in healthcare settings separately with the unmet need for GPs and with the unmet need for nurses. In addition, both models (perceived unjust treatment with the unmet need for GPs and with the unmet need for nurses) were separately adjusted for age, gender, marital status, education, employment, and self-rated health. The odds ratios (OR) and corresponding 95% confidence intervals (95% CI) were calculated.

Weights were used to calculate the prevalence of need for primary care, of perceived treatment and of self-rated health in all logistic regression analyses. The data were weighted by gender and age (5-year age groups) to match the general population of Finland on 31 December 2017 [48]. All analyses were performed using the SAS 9.4 statistical software (SAS, Helsinki, Finland).

## 3. Results

The characteristics of the study population are presented in Table 1. More than half of the respondents were female (61%), and 41% of respondents were 30–54 years old. Of the male respondents, 47% were either single, divorced or widows, while the corresponding proportion of women was 63%. Secondary or higher education had 41% of the male and 39% of the female respondents. Almost one-third of the respondents were unemployed. 

The prevalence of the need for primary care, of the unmet need for health services and of the perceived unjust treatment, as well as the self-rated health among the Finnish Roma, are presented in Table 2. The male respondents reported a need to consult a health centre GP or nurse during the last 12 months less often than the female respondents (GPs: 73% vs. 85%; nurses: 67% vs. 72%). Of those who had needed GP services, 32% of men and 31% of women felt that their need had not been met. For those who had needed the primary services provided by nurses within the past 12 months, the prevalence of unmet needs was 21% among men and 27% among women. Around 20% of the males and 13% of the female respondents had experienced unjust treatment in healthcare settings. Moderate or lower self-rated health was reported by 47% of the Roma men and 50% of the Roma women. 

The association between the perceived unjust treatment and the unmet need for GPs is presented in Table 3. Those who had experienced unjust treatment in healthcare had higher odds (OR = 6.83; *p* < 0.001) for unmet need for GPs than those who had felt they had been treated justly. This association was robust (OR = 6.44; *p* < 0.001) after adjustment for gender, age, level of education, employment status, marital status and self-estimated health. The Finnish Roma who were single or divorced and with a lower level of education were more likely to experience unmet need for GP. Age, gender, employment status, and self-rated health were not associated with the perceived unjust treatment or the unmet need for GP services.

The final model explained 29% of the variation in the Finnish Roma unjust treatment in GP services.

The association between the perceived unjust treatment and the unmet need for services provided by nurses is presented in Table 4. The Finnish Roma who felt that they had been treated unjustly in healthcare settings had also higher odds for the unmet need for services provided by nurses (OR = 11.18; *p* < 0.001) than those who felt that they had been treated justly. This association increased further (OR = 12.73; *p* < 0.001) after adjustment for gender, age, education, employment status, marital status, and self-estimated health. The other tested factors (age, gender, marital status, education, employment status, and self-rated health) were not associated with the perceived unjust treatment or the unmet need for services provided by nurses. The final model explained 35% of the variation in the perceived unjust treatment in services provided by nurses experienced by the Finnish Roma.

The Finnish Roma representatives agreed that there is definitely unjust, even stereotype-based treatment in the healthcare settings [43]. The representatives also brought up and emphasized the diversity of the phenomenon, and some of the factors behind such experiences were also explained. Perceived unjust treatment may arise from the patient’s previous experiences in similar settings, as well as their health status and the patient’s own expectations of the treatment and cure. A practical example was given by the representatives: the patient comes to an appointment with certain expectations regarding their treatment. The GP or nurse might provide the patient with a basic level of care and might not consider it necessary to refer the patient to the specialized medical care. The disagreement over the level of care needed may become a difference of opinion between the GP or nurse and the patient, and this may result in the experience of unjust treatment. The patient may assume that the underlying cause of the disagreement is the patient’s ethnic background, even though the treatment may be no different from that received by other service users. Thus, the experience can be subjective, but it should not be underestimated. Nevertheless, it is important that researchers dare to critically assess the cause, effect and underlying factors behind the perceived unjust treatment.

## 4. Discussion

Of the Finnish Roma who had needed primary care services, nearly one-third (30%) had experienced unmet needs for GP services while one-fifth (24%) had experienced unmet needs for services provided by nurses. Corresponding proportions for the general population has been found to be considerably lower (16% for GPs and 10% for nurses) by a previous study [49]. The same study found the prevalence for the need for GP services among the general population to be 72%, while this study found the corresponding prevalence among the Finnish Roma to be 79%. Although the results for the general population and the Finnish Roma are not directly comparable due to differences in data collection methods, the results imply a clear discrepancy in unmet need for primary care between the general population and the Finnish Roma. Several previous studies have suggested that ethnic minorities are more likely to experience unmet need for healthcare services compared to the general population (e.g., [4,32,50,51]). 

Single, divorced, and widow respondents as well as those with lower education experienced unmet need for GP services more often than those in a relationship and with higher education. This result is analogous to previous studies that have examined how marital status and lower education are connected to the unmet need for healthcare (e.g., [52,53,54]). Surprisingly, in this study self-rated health was not associated with unmet need for primary care provided by GPs or nurses among the Finnish Roma. This result differs from several previous studies that have observed that those who express lower self-rated health are more likely to experience unmet need for care and have problems accessing healthcare [32,55]. This result may be explained by the custom among the Finnish Roma of not easily seeking treatment because they believe that a person is healthy unless illness or discomfort causes undue inconvenience. The older Roma, in particular, may avoid seeking healthcare services [56]. However, the fact that the Roma do not eagerly seek health-related services does not necessarily mean that the perceived need for care is reduced. Moreover, low socio-economic status, such as lower education, has been associated with several negative health behavioural patterns, such as a lack of physical exercise and a high prevalence of smoking and chronic diseases, as well as their risk factors [3,33]. In the case of the Finnish Roma, this does not automatically mean that a person experiences a subjective need for healthcare due to the cultural habits.

In this study, a strong association was observed between those reporting unmet need for primary care provided by GPs and nurses and those who reported perceived unjust treatment. Those who felt they had been treated unjustly were six times more likely to experience an unmet need for GP services and as much as twelve times more likely to experience an unmet need for services provided by nurses. The results of this study contrast with those of Seale et al. (2005) regarding the differences between nurses and GPs. Seale et al. (2005) examine the reason why patients are usually more satisfied with nurse consultations than consultations by GPs [57]. Their study suggests that nurses spend twice as long a time with their patients and talk more about the treatment and the social and emotional aspects of patient lives compared to GPs, and this discrepancy results in higher satisfaction with the services provided by nurses. This explanation is not verifiable by our data, because the questionnaire used did not differentiate between the reasons for satisfaction for the two types of services. At some health centres, and in some cases (e.g., on-call visits), nurses act as gatekeepers to the GP services, which may explain the higher association between unjust treatment and unmet needs for services provided by nurses compared to those provided by GPs. There may also be a difference in the needs of patients seeking services GP services and services provided by nurses. In some cases, GP consultations may be considered necessary because of a need for a prescription or further treatment in specialized care. Moreover, the Roosa study report has found that the Finnish Roma have a higher overall need for GP services compared to the need for services provided by nurses [23].

It is well known that the rate of experienced discrimination is high among the Finnish Roma [7]. This study found that 17 percent of the Finnish Roma had experienced unjust treatment in healthcare settings. A previous study that used the same data showed the prevalence for perceived unjust treatment to be even higher other public services: for example, one-fifth of the Finnish Roma men and one-fourth of the Finnish Roma women had experienced unjust treatment in social services [7]. Perceived discrimination is common among the Roma, and other ethnic minorities in many other European countries, as well [58,59]. 

Perceived unjust treatment can be discrimination, and as such may affect a person’s health and wellbeing. Those who have felt that they have been treated unjustly or been discriminated against often report more physical and mental health problems than others [2,8,60,61,62]. Roma ethnicity has been found to be strongly associated with poor self-rated health status and high rates of chronic diseases in many European countries [3,33]. In addition to discrimination and perceived unjust treatment, a poor socio-economic status, unhealthy lifestyle, and potential barriers precluding access to healthcare among the Roma population have been suggested to potentially lead to health problems (e.g., [3,63]). 

As recognized by the Finnish Roma, perceived unjust treatment in healthcare settings is a complex, multifaceted question. The results of this study can be discussed from individual, cultural and social point of view. Discrimination and perceived unjust treatment can be seen as a subjective experience, influenced by a multitude of factors such as previous experiences, attitudes or the communication between the health worker and the patient seeking care. Furthermore, the patient’s lack of trust towards the healthcare system, health-illiteracy or socio-economic factors may partly explain the experiences of unjust treatment [64,65]. Culturally, the Roma have a system of symbolic cleanliness and hierarchy system, in which healthcare settings like hospitals are perceived as ‘dirty places’. In addition, the Roma culture is communal, and being the only representative of one’s own culture in a healthcare setting can feel frightening and this fear may lead to a will to avoid those situations [66]. 

In order to fully understand the phenomenon, structural and social aspects need also be considered. Recurring experiences of unjust treatment in welfare services may indicate an institutionalized form of discrimination in a society. In Finland as well as in some of the other Nordic countries, an attempt has been made to intervene in structural discrimination through policy programs [67,68,69]. These programs seek to promote human and minority rights and support minorities in maintaining their own culture and language. However, Helakorpi et al. [70] has maintained that, while trying to increase the school attendance among the Roma children, policy programs have sometimes problematized the Roma and their relationship to school rather than concentrated on the structures and practices that produce the inequality in education. There is a risk that the well-meaning policy programs of equality may miss their opportunity to intervene in structures and practices hence making it difficult to address the root causes of inequality and the way it is reproduced in a society. 

Several studies and policy programs recommend reducing discrimination and perceived unjust treatment by improving bidirectional guidance between the Roma and the service providers (e.g., [71,72]). For instance, the municipality of Oslo in Norway recommends building a guidance service for the Roma to ensure that information about services is available specifically for them. Furthermore, it should be ensured that the health service providers have competence in and knowledge about the Roma culture, identity and way of life [73]. Miranda et al. [74] suggest promoting Roma health justice by local projects that would make Roma voices heard—both individually and collectively. The aim is to provide services that are formed and maintained by the community itself. This kind of a community-based approach could ensure a collective ownership of the services and thus create a network built on trust (see also [75,76]). It has also been shown that gatherings organized by Roma women themselves, have an impact in the individual level to their living conditions, while in the societal level the gatherings enhance women’s associational life resulting in a new kind of mobilization [77].

The following implications follow from our research. Firstly, unjust treatment in healthcare must be critically assessed, taking into account the healthcare system’s possible discriminative practices, structural discrimination and social values, norms, and attitudes associated with the healthcare system. Discrimination should be reduced by informing the healthcare staff about the Roma culture, by increasing the easy everyday encounters between the different groups and by an active interference in the notable disadvantages. In the future, what should be investigated is how the patient’s lack of trust towards the healthcare system, health-illiteracy, or socio-economic factors may explain the experiences of unjust treatment. In addition, details of the unjust treatment and a question of adequate treatment are relevant topics to research. Secondly, the Roma community’s own resources should be strengthened, supported, and utilized. The Roma and other marginalized communities need to be involved in developing the health services. In the future, the now sparse research tradition among (and with) Finnish Roma should be extended. Participatory research methods should be improved further, possibly by taking into account the mix-method nature of the research. Furthermore, the health situation of the Roma women should be more closely examined in the further research in the field.

### Study Limitations

In this study, the selection of the target group was based on self-identification as a random sample of an ethnic minority is not possible to obtain due to the Personal Data Act (523/1999, §6). Self-identification is also mentioned in the Finland’s National Roma Policy Program [22] as a form of defining the Roma ethnicity. Thus, the respondents were recruited by a snowball sampling method and all those identifying themselves as Finnish Roma were able to participate in the study. The number of respondents in this study was rather low (*N* = 365) in comparison to estimated numbers of the Finnish Roma, and there is no conclusive way of knowing how the respondents represent the rest of the Finnish Roma population. These limitations mean that the results cannot be generalized and the results are mainly indicative. In any case, the study and the results can be seen as an important first step to study this minority group, which can be demanding to reach. In future studies, it is advisable to improve the process of motivating and recruiting participants, which may be easier after this study has shown that no harm was caused to the participants or to the Roma community. 

Some factors increased the validity of the results. The data was collected in many different ways (health examination, a structured face-to-face interview or a self-filled questionnaire) and the participants were persons from all socioeconomic backgrounds, age groups, and most regions of Finland. Versatility allowed different people to participate in the study. Moreover, the involvement of representatives of various Roma organizations in the interpretation of the results improved the validity of the study. 

The data was collected by the structured questionnaire and the dimensions of treatment are not known. The differences between unconscious and conscious bias as well as between real and perceived discrimination cannot be studied with this data. This would require, for example, ethnographic research. 

## 5. Conclusions

This study examined the role of perceived unjust treatment in healthcare settings in explaining self-reported unmet needs for services provided by GPs and nurses among the Finnish Roma. Perceived unjust treatment in healthcare settings were important predictors of the unmet needs for services provided by GPs and nurses. In addition, it was found that the single or divorced, and those with lower education, were more likely to experience more unmet needs for GPs than those in a relationship and those with higher education.

## Figures and Tables

**Table 1:** The descriptive statistics of the study population, %, *n*.

Variable		Male	Female	Total
		*n* = 142	*n* = 223	*n* = 365
		%	%	%
Age	18–29 years	29	27	28
	30–54 years	40	42	41
	55+	31	31	31
Marital status	Single *	47	63	55
Education	Secondary or higher	41	39	40
Employment status **	Employed	18	22	20
	Unemployed ***	31	32	31
	Other ****	51	46	48

* includes singles, the divorced and widows; ** limited to working aged (18–64-year-old respondents); *** includes the unemployed and the retired; **** includes students and those on family leave.

**Table 2:** The prevalence of the primary care need, of the unmet need, and of the perceived unjust treatment and the self-rated health, %, *n*.

Variable		Male	Female	Total
		*n* = 101 *–142	*n* = 196 *–223	*n* = 297 *–336
		%	%	%
Healthcare need for General Practitioner (GP)	-	73	85	79
Healthcare need for nurse	-	67	72	70
Unmet need for GP **	-	32	31	31
Unmet need for nurses **	-	21	27	24
Perceived unjust treatment:	Treated unjustly	20	13	17
	Treated justly	80	87	83
Self-rated health:	Moderate or lower	47	50	49
	Good or rather good	53	50	51

* The small number of observations is because the question is presented only on a short questionnaire; ** of those who had needed the services.

**Table 3:** The association between the perceived unjust treatment and the unmet need for GPs, %, 95% CI and *p*-value. The results are adjusted for age, gender, marital status, education, employment status, and self-rated health **.

Variable		OR	95% Cl	*p*-Value
Perceived unjust treatment *	Treated unjustly	6.83		
	Treated justly	1	3.45–13.55	<0.001
Perceived unjust treatment **	Treated unjustly	6.44		
	Treated justly	1	2.93–14.14	<0.001
Age	18–29 years	1		
	30–54 years	2.53	0.88–7.26	0.083
	55+	1.10	0.36–3.43	0.864
Gender	Male	1		
	Female	0.98	0.50–1.89	0.946
Marital status	Single or divorced	2.06	1.00–4.23	0.049
	Couple relationship	1		
Education	Secondary or higher	1		
	Primary or lower	3.74	1.50–9.33	0.005
Employment status	Employed	1.00		
	Unemployed	0.66	0.20–2.23	0.502
	Other	0.90	0.30–2.68	0.850
Self-rated health	Moderate or lower	1.91	0.96–3.82	0.067
	Good or rather good	1		

* no adjustments; ** adjusted for confounders.

**Table 4:** The association between perceived unjust treatment and unmet need for nurses, %, 95% CI and *p*-value. The results adjusted for age, gender, marital status, education, employment status, and self-rated health **.

Variable		OR	95 % Cl	*p*-Value
Perceived unjust treatment *	Treated unjustly	11.18		
	Treated justly	1	5.07–24.67	<0.001
Perceived unjust treatment **	Treated unjustly	12.73		
	Treated justly	1	4.94–32.80	<0.001
Age	18–29 years	1		
	30–54 years	3.21	0.94–10.99	0.064
	55+	0.82	0.20–3.35	0.787
Gender	Male	1		
	Female	1.48	0.63–3.47	0.370
Marital status	Single or divorced	1.66	0.70–3.95	0.253
	Couple relationship	1		
Education	Secondary or higher	1		
	Primary or lower	2.82	0.95–8.39	0.063
Employment status	Employed	1.00		
	Unemployed	1.02	0.24–4.26	0.979
	Other	1.01	0.25–4.16	0.987
Self-rated health	Moderate or lower	0.88	0.37–2.12	0.781
	Good or rather good	1	-	-

* no adjustments; ** adjusted for confounders.
